# Microbial Communities in a Flow-Through Fish Farm for Lumpfish (*Cyclopterus lumpus* L.) During Healthy Rearing Conditions

**DOI:** 10.3389/fmicb.2019.01594

**Published:** 2019-07-12

**Authors:** Irene Roalkvam, Karine Drønen, Håkon Dahle, Heidrun Inger Wergeland

**Affiliations:** ^1^Department of Biological Sciences, University of Bergen, Bergen, Norway; ^2^K.G. Jebsen Centre for Deep Sea Research, University of Bergen, Bergen, Norway

**Keywords:** lumpfish, *Cyclopterus lumpus*, flow-thorough, microbial community, amplicon, fish farm, aquaculture, fish pathogens

## Abstract

Lumpfish can efficiently remove sea lice from Atlantic salmon in net-pens, and production of lumpfish in closed fish farms is a new, fast developing industry in Norway. However, periodic outbreaks of bacterial diseases in the fish farms represent a large problem, both economically and ethically. Therefore it is important to obtain a better understanding of how microbial communities develop in these production facilities. Knowledge on the characteristics of microbial communities associated with healthy fish could also enable detection of changes associated with disease outbreaks at an early stage. In this study we have monitored microbial communities in a fish farm for lumpfish during normal operational conditions with no disease outbreak by using 16S rRNA gene amplicon sequencing. The study involved weekly samplings of water and biofilms from fish tanks, and fish. The results revealed that the microbial communities in fish tank water were different from the intake water. The water and biofilm in fish tanks were highly similar in regards to microbial community members, but with large differences in relative abundances for some taxa. The sampled fish were associated with mostly the same taxa as in tank water and biofilm, but more variation in relative abundances of different taxonomic groups occurred. The microbial communities in the fish farm seemed stable over time, and were dominated by marine bacteria and archaea within *Alphaproteobacteria*, *Epsilonproteobacteria*, *Flavobacteria*, *Gammaproteobacteria*, *Thaumarchaeota*, *Planctomycetes*, *Sphingobacteriia*, and *Verrucomicrobiae* (>10% relative abundance). Bacterial genera known to include fish-pathogenic strains were detected in all types of sample materials, but with low relative abundances (<5%). Exceptions were some samples of fish, biofilm and water with high relative abundance of *Tenacibaculum* (<85.8%) and *Moritella* (<82%). In addition, some of the eggs had a high relative abundance of *Tenacibaculum* (<89.5%). Overall, this study shows that a stable microbial community dominated by various genera of non-pathogenic bacteria is associated with a healthy environment for rearing lumpfish. Taxa with pathogenic members were also part of the microbial communities during healthy conditions, but the stable non-pathogenic bacteria may limit their growth and thereby prevent disease outbreaks.

## Introduction

The production of lumpfish in Norway has increased over the last 5 years, with a total production of approximately 26 million fish in 2017, worth 469 million NOK (€ ∼49.2 million) (Norwegian Directorate of Fisheries, 2017). The cleaner fish co-inhabit the net pens with the salmon where they remove lice from the fish’s skin. Given optimal conditions, cleaner fish are very efficient and can consume several hundred sea lice per day from infected salmon (Imsland et al., 2014; Powell et al., 2018). The requirement for cleaner fish is estimated to reach 50 million individuals by 2020, according to Powell et al. (2018), of which most will be bred lumpfish. Hence, rearing lumpfish in land-based, closed fish farms represents an industry with high profit margin and increasing demand for production efficiency and high quality. Among the different species of cleaner fish, lumpfish is the best suitable species for fish farming. This is due to the good access to wild eggs and milt, wide and low temperature tolerance in fry and fish, social and non-territorial behavior, and excellent growth and development when fed with pellets instead of live fish feed (Treasurer, 2018).

Successful rearing of lumpfish in fish farms is dependent on many factors, such as water quality, temperature and microorganisms present in the facility that could affect the water quality and influence the fish health. Although vaccine programs for lumpfish are presently developing, the fish pathogens *Aeromonas salmonicida*, *Vibrio anguillarum*, *Vibrio ordalii*, *Pseudomonas anguilliseptica*, and *Pasteurella* sp. are still the main cause of infections and economical losses in lumpfish farms (Norwegian Veterinary Institute [NVI], 2017). Microbial studies on lumpfish in rearing facilities have mainly focused on pathogenic bacteria that could cause infections and disease outbreaks (Powell et al., 2018; Scholz et al., 2018). Others have focused on the bacteria present during larvae development (Dahle et al., 2017); however, the average lumpfish spend only a short period as larvae in the fish farm during a normal production cycle. To our knowledge, this is the first study on the non-pathogenic members of the microbial communities in a water flow-through rearing system for lumpfish; and how these communities change over time or in response to environmental variations. Furthermore, the mechanism for increased amounts of opportunistic pathogenic bacteria and subsequent disease outbreak is not fully understood.

The aim of the study was to characterize microbial communities present at different sites in a flow-through fish farm for lumpfish during healthy conditions. Weekly sampling was performed over a 5 months period, during a rearing cycle with standard water quality, normal fry development and no disease or infections in the fish. By using 16S rRNA gene sequencing, we identified possible probiotic microorganisms and obtained detailed knowledge about distributions of microorganisms, including how their relative abundances vary in different parts of the rearing facility and how they change over time. This study provides knowledge about the microbial communities present at healthy rearing conditions, allowing the recognition of potential changes in the community structures associated with disease outbreaks.

## Materials and Methods

### Sampling

**FIGURE 1:**
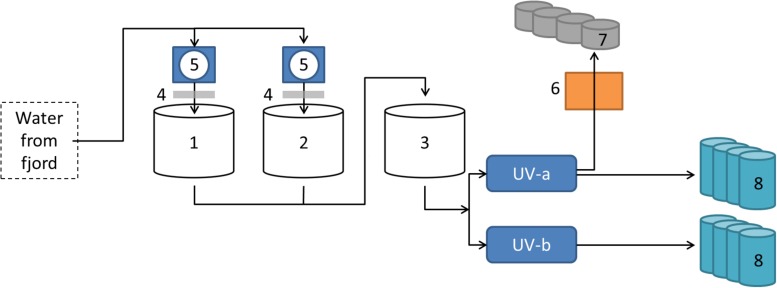
Overview of intake water at the Vest Aqua Base. Water originated from ∼70 m depth in the adjacent bay, and was stored in three land-based tanks (1–3). Storage tank 1 and 2 were also connected to degassing columns (4) and drum filters (5). The water was disinfected by UV treatment in two pipe lines (UV-a and UV-b) before entering the facility. A separate line had an additional sand filter (6) before entering the hatchery (7). Water from both UV-treated pipe lines supplied parallel fish tanks in the weaning- and on-growing sections (8).

The sampling site in this study was the flow-through fish farm of Vest Aqua Base AS, located at Årskog at Fitjar in Norway. The intake water originated from ∼70 m depth in Fitjarvik, the adjacent marine bay. The water was stored outside in three large tanks, where associated drum filters and degassing columns improved the water quality. The water inlet was split into two pipelines with independent UV treatment for disinfection (Figure 1). The facility included a hatchery, which had an additional sand filter for water treatment, a section with weaning tanks and a larger area with grow-out tanks. Weekly sampling was performed in the period April-August 2017, in addition to eggs sampled in February 2017. Of the 15 weeks of sampling, the first 4 weeks included material from eight parallel tanks from the weaning section, while the last 11 weeks included material from four larger tanks in the on-growing section. The rearing conditions for the weaning section were flow rate of 10–16 L/min in 550 L tanks, and a temperature of 7.6–8.0°C. The on-growing section had 2500 L tanks with a flow rate of 20–60 L/min and temperature of 7.7–11.1°C.

From each tank, 240 ml water was filtered using 0.22 μm Sterivex filter units (Merck) in order to collect all microorganisms. For biofilm harvest, several 4.8 mm × 200 mm plastic (nylon 66 material) cable ties rinsed in 70% ethanol were placed in each tank; with the ties protruding in a circle formation from a central cable attached to a water pipe in the tank. The colonization and development of biofilms could be followed over time by weekly removal of cable ties. Biofilm harvested with swabs directly from the tank wall were included for comparisons with the cable ties. The eggs were sampled using sterile tweezers, and stored in RNA-Later. The sampled fish were 3–22 mm in length. Each sample comprised whole animals, typically 4–5 fry or 1–2 fish, depending on size. Fish were sampled and stored in RNA-Later as intact fish. All samples were kept cool during transportation to the lab at University of Bergen, where they were stored at −20°C until further analyses.

The material sampled included 75 samples of fish, 76 samples of water from fish tanks, 62 samples of biofilm from cable ties, and 55 additional samples with material such as biofilm from tank walls, drain pipes, dead eggs or fish, water storage tanks, drum filter tanks and UV treatment system (see Supplementary Table S1 for sample overview). In addition, 12 samples of eggs from the hatchery and eight samples of egg from incubators in the weaning section were included.

### DNA Extraction

DNA was extracted from the samples using kits designed for the different material types: DNeasy Power Water kit (Qiagen) was used for Sterivex filters, as suggested by the manufacturer. The plastic casing of the filter was cracked open using a pair of tongs and the filter material was removed from the plastic core using sterile scalpel. DNeasy Power Biofilm kit (Qiagen) was used for DNA extractions from biofilms. The cable tie was cut into 4 pieces, which could fit into the bead tubes supplied by the kit. After incubation with the lysis buffer, the biofilm material had detached from the cable ties and these were removed from the tube. Bead beating was performed in a FastPrep 24 instrument (MP Biomedicals) at 5 m/s for 30 sec. The rest of the protocol was in accordance with the manufacturer’s suggestions. The High Pure PCR Template Preparation kit (Roche) was applied to both eggs and fish. Due to the small size, several eggs or whole fish were included in the DNA extraction protocol. The protocol 2.4: “Isolation of nucleic acids from mammalian tissue” and protocol 2.8: “Protocol for washing and Elution” were followed as suggested by the manufacturer. Lysis was complete after 60–90 min, depending on material and sample size.

### Amplicon Library Preparation and Sequencing

The 16S rRNA genes amplicon libraries were prepared in a two-step PCR in order to minimize PCR biases introduced by the barcoded primers, as suggested by Berry et al. (2011). In the first PCR, samples were amplified using the universal primers 519f (5′-CAGCMGCCGCGGTAA) (Ovreas et al., 1997) and 805r (5′-GACTACHVGGGTATCTAATCC) (Klindworth et al., 2013). The reactions contained 1 × HotStarTaq master mix (Qiagen), 1 mM of each primer and sample DNA. The thermal program included 15 min activation of the Taq enzyme at 95°C, followed by 25–32 cycles of gene amplification, i.e., 30 sec at 94°C, 30 sec at 56°C and 90 sec at 72°C. The final elongation was done at 72°C for 7 min. Triplicates of each sample were pooled, and then visualized and assessed by 1D gel electrophoresis. PCR products were purified using Agencourt AMPure XP beads (Beckman Coulter), with a 0.7 volume ratio between AMPure reagent and PCR product. The protocol followed was supplied by Beckman Coulter. Samples were quantified using Quantus Fluorometer (Promega Corporation). In the second PCR, the reactions contained 1x HotStarTaq master mix (Qiagen), 1 mM of each primer and approximately 100 ng template, i.e., PCR product from the first reaction. The primers were barcoded and adapted to the sequencing technology used, and the forward primer includes an individual tag for sample identification. The thermal program was the same as before, however, only seven cycles of amplification was used. After purification with Agencourt AMPure XP beads, quantification using Quantus Fluorometer (Promega Corporation) and visualization using 1D gel electrophoresis; 96 samples were pooled in equimolar concentration and the pool diluted to 40 pM. In total, three amplicon libraries comprising 281 samples were sequenced at the University of Bergen, Norway using Ion Torrent Personal Genome Machine (PGM) technology.

### Bioinformatics

The down-stream 16S rRNA gene sequence analysis included the following steps: Sequences were filtered and clustered into operational taxonomic units (OTUs) using USEARCH (Edgar, 2010) and UPARSE (Edgar, 2013). Quality filtering and trimming to 250 bp was performed with the “-fastq_filter” command using options “-fastq_trunclen 250,” and “-fastq_maxee 1.” Chimeric sequences were detected and removed with the “-uchime_ref” command using the Gold database as reference^1^. *De novo* OTU clustering was performed with a cut-off of 97% nucleotide sequence similarity using the “cluster_otus” command. Taxonomic classification was performed in QIIME (Caporaso et al., 2010), using the command “summarize_taxa_through_plots.py” and the Silva version 128 as reference database (OTU table is available in Supplementary Table S2). Most of the sequences could be taxonomically classified to genus level, and the data were presented as relative abundance in order to make the microbial communities in different samples comparable. Sequences classified to a higher taxonomical level were pooled and shown as “Unclassified at genus level” in this study. Genera with names without standing in nomenclature in the Silva database were presented at a higher taxonomical level. Sequences are available at Sequence Read Archive (SRP150702) under Bioproject PRJNA476040 and Biosample SAMN09425395.

### Statistical Analyses

The analyses were performed on data at genus level with values given as relative abundance, excluding 5 of the 281 samples due to low number of reads. The principal coordinates analysis (PCoA) was performed using the “Vegan” package in R version 3.5 (Oksanen et al., 2018; R Core Team, 2018), using the “wcmdscale” function with Bray-Curtis as dissimilarity index. We used PERMANOVA, as implemented in the “adonis” function (Anderson, 2001) in Vegan, to test for significant differences between types of sample material. As we did not find any clear evidence for community changes over time, repeated samples from the same type of sample material were considered as time independent replicates. The null hypothesis of the PERMANOVA test is that the metric centroid is the same in different sample types (Anderson and Walsh, 2013). When performing pairwise comparisons of multiple sample types, “adonis” was run on corresponding subsets of the whole dataset. The reported *p*-values were adjusted with the “fdr” correction (Benjamini and Hochberg, 1995), as implemented in the function “p.adjust” in Vegan. In order to test for differences in dispersion, with the null hypothesis that the average dispersion within sample types is the same in all types of sample material (Anderson and Walsh, 2013), we used PERMDIST (Anderson, 2006), as implemented through the “betadisper” function in Vegan. The default number of permutations (999) was applied when using both “adonis” and “betadisper.” Hierarchic cluster analysis of samples was done based on a Bray–Curtis distance matrix and using the ward algorithm (ward.D2) in R (Murtagh and Legendre, 2014; see Supplementary Table S3 for distance matrix of all samples).

## Results

This study on development of microbial communities in a closed fish farm for lumpfish was based on weekly sampling in the period April-August 2017 at the flow-through facility at Vest Aqua Base AS, located on the west coast of Norway. Sample collection included eggs sampled from the hatchery and from incubators in the weaning section, whole fish, water from fish tanks, biofilm from cable ties and swabs of tank wall, and additional sample material such as swabs in drain pipes, and water from water storage tanks, drum filter and UV treatment system (referred to as intake water hereafter). The fish were kept 4 weeks in the weaning section, where we monitored eight parallel tanks, followed by 11 weeks in the on-growing section, where four parallel tanks were sampled regularly. In total, approximately 12.3 million reads were retrieved using the Ion Torrent sequencing technology (Table 1). After filtration and data clean-up, each sample had on average 32516 reads, which were clustered into operational taxonomical units (OTUs).

**TABLE 1:** Data statistics for filtering and OTU clustering of amplicon reads.

**Data**	**Total**	**Average for each sample**
Number of samples	281	–
Number of reads	12,320,353	43,078
Number of reads removed^*^	3,020,625	10,561
Number of reads for OTU analysis	9,299,728	32,516
Number of OTUs	14,415	–

^*^The reads removed due to inadequate length or poor quality constitutes 8–22% of reads in the samples.

### Community Analysis

**FIGURE 2:**
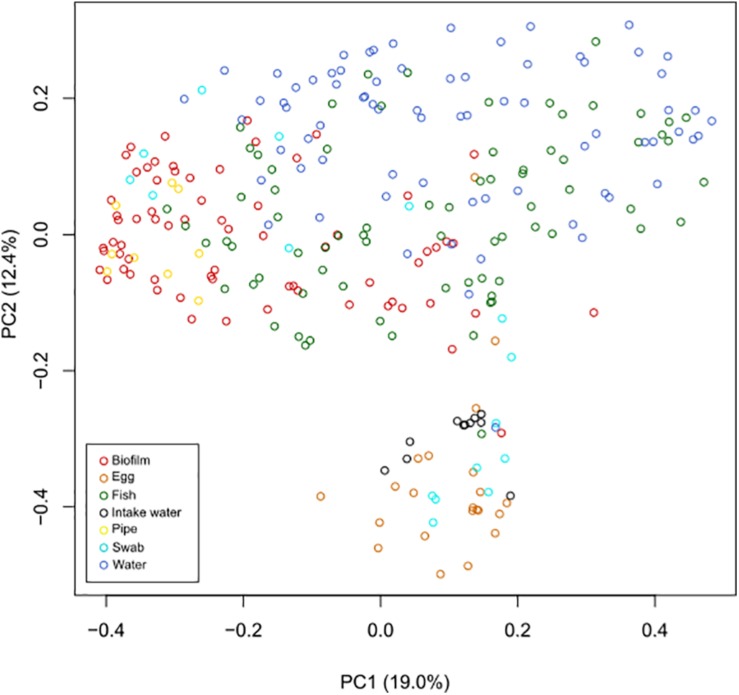
Principal coordinates analysis (PCoA) performed on all samples at genus level. Samples were compared using Bray–Curtis distance matrix, and the similarities were visualized in a PCoA plot. Samples of intake water and most egg samples formed a separate cluster, together with a few other samples. In addition, sampled tank water and samples comprising biofilms (from cable ties, tank walls, and drain pipes) also seemed to cluster separately, while sampled fish were more evenly distributed in the plot.

An initial hierarchal cluster analysis of all samples on genus level revealed that the data could not be easily distinguished in separate clusters based on different types of sample material or different sample locations (Supplementary Figure S1). A Principal Coordinates Analysis (PCoA) was performed on data from all samples on genus level in order assess variations in community structure between and within different sample categories. All samples of intake water and most sampled eggs formed a distinct cluster, which also included a few other samples (Figure 2). Samples from fish tank water seemed to be separated from samples comprising biofilms, while samples of fish were widely dispersed. A permutational multivariate analysis of variance (ADONIS) supported the PCoA results, showing that centroids of the sample groups indicated in Figure 2 were not the same (*F* = 15.2, *p* < 0.01, Df = 275). Samples from fish tank water seemed to be separated from samples comprising biofilms, while samples of fish had a more even distribution in the plot. ADONIS analyses on each sample group (using adjusted *p*-values) further revealed that intake water samples were significantly different to any other sample group (*p* < 0.01) (Table 2). However, it should be noted that the ADONIS results could be influenced by unbalanced sampling, as well as differences in dispersion, which were found to be significantly different using the “betadisper” function in Vegan (*F* = 11.3, *p* = <0.01).

**TABLE 2:** The permutational multivariate analysis of variance (ADONIS) between different types of sample material.

		**Adjusted *P*-values and R^2^**					
		**Egg**	**Intake water**	**Fish**	**Drain pipe**	**Swab of tank wall**	**Fish tank water**
Biofilm	P	0.0011	0.0011	0.0011	0.0030	0.0011	0.0011
	R^2^	0.1908	0.2353	0.1090	0.0460	0.0683	0.1789
Egg	P		0.0011	0.0011	0.0011	0.0011	0.0011
	R^2^		0.2914	0.1435	0.3098	0.1367	0.2343
In. water	P			0.0011	0.0011	0.0011	0.0011
	R^2^			0.1509	0.5180	0.2878	0.2020
Fish	P				0.0011	0.0011	0.0011
	R^2^				0.0801	0.0466	0.0874
Pipe	P					0.0021	0.0011
	R^2^					0.1990	0.1466
Swab	P						0.0011
	R^2^						0.1092

### Microbial Communities in Water and Biofilm

**FIGURE 3:**
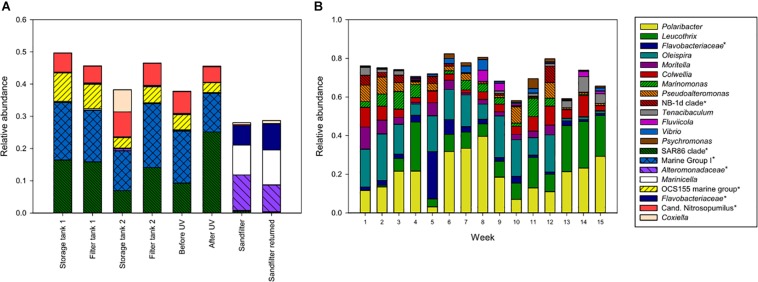
Amplicon libraries of intake water **(A)** and water from fish tanks **(B)**. Intake water included storage tanks, drum filter tanks, and water before and after UV treatment, in addition to the sand filter connected to the hatchery. For fish tanks, the bars show the average community compositions from 8 parallel weaning tanks sampled in week 1–4, and 4 parallel grow-out tanks sampled in week 5–15. Taxa with >5% relative abundance in at least one sample are shown. This includes unnamed genera in the Silva database, indicated with an asterisk and named at a higher taxonomic rank.

The microbial communities in different parts of the intake water, i.e., seawater before entering the fish tanks, included both *Archaea* and *Bacteria*, where 37–49% of the microbial communities comprised taxa within the SAR86 clade, OCS155 marine group, *Coxiella*, “*Candidatus* Nitrosopumilus” and other members of Marine Group 1 (Figure 3A). The UV-treated water also supplied incubators in the hatchery through a separate pipeline containing a sand filter (Figure 1), which delivered water with a different community structure to the eggs in the hatchery. This water was enriched in various bacterial taxa, and was dominated by members of *Alteromonadaceae* and *Flavobacteraceae*, and *Marinicella* (Figure 3A). Furthermore, the microbial communities in water from the fish tanks were different from the intake water, and were dominated by members of *Bacteria* only (Figure 3B). Water from the eight weaning tanks and four grow-out tanks displayed similar community structures over time. The exceptions were a decrease in relative abundance for some of the dominant taxa during the last 3 weeks of sampling, and the clear change in community composition observed in week 5, which was probably related to transferring the fish from weaning tanks to the larger grow-out tanks (Figure 3B).

Biofilms formed quickly on tank walls and pipes, and daily cleaning routines were implemented for keeping the biofilm development at a minimum. Thicker biofilms were found inside the drain pipes where cleaning was difficult. In order to harvest a comparable surface area with biofilm weekly, several cable ties were placed in the fish tanks simultaneously and then removed one at a time. Biofilms from the cable ties were also compared to swabbed areas from each tank wall, in order investigate if differences in the surface material influenced biofilm community structure. The dominating taxa in biofilms from swabbed samples were very similar to biofilms from cable ties (Figure 2), regarding presence of taxa and their relative abundances (data not shown). Hence, the cable ties represented the tank wall in a satisfactory manner, and were thus considered as a good method for studying biofilm establishment and development.

**FIGURE 4:**
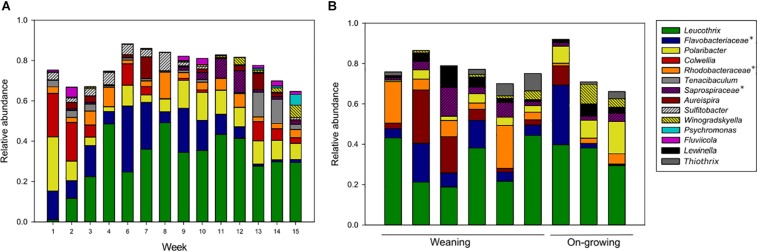
Amplicon libraries of biofilm sampled in fish tanks **(A)** and drain pipes **(B)**. The average community structures from fish tank samples were studied in 8 parallel weaning tanks sampled in week 1–4, and 4 parallel grow-out tanks sampled in week 6–15. The drain pipes were sampled only once and included six samples from the weaning section and three samples from the on-growing section. Taxa with >5% relative abundance in at least one sample are shown. This includes unnamed genera in the Silva database, indicated with an asterisk and named at a higher taxonomic rank.

Biofilms on cable ties were sampled weekly from all fish tanks. The only exception was the first week in the grow-out tanks (week 5), when the cable ties were installed. Biofilms comprising similar community structures established in tanks from both weaning section and on-growing section in the fish farm (Figure 4A). The frequent sampling revealed that the taxa *Leucothrix* and members of *Rhodobacteraceae* showed a trend of increasing relative abundance over time. Contrarily, the high relative abundance of the taxa *Polaribacter* and *Colwellia* found shortly after colonization of a clean surface decreased over time.

Comparisons of the microorganisms in water and biofilms showed that the relative abundances for some of the taxa were different. The water samples were abundant in *Oleispira*, *Pseudoalteromonas*, *Marinomonas*, NB-1d clade, and *Vibrio*, which had a relative abundance of <5% in the biofilms. In contrast, the taxa *Leucothrix*, members of *Rhodobacteraceae* and *Saprospiraceae*, *Aureispira*, and *Winogradskyella* were only found with a relative abundance >5% in biofilms, not in water samples. Apart from these taxa, many of the abundant microorganisms were present in both sample types, and seemed to be ubiquitous in the aquaculture environments.

Biofilms from inside the drain pipes were collected in order to compare the communities in an area where cleaning and proper maintenance can be difficult to obtain. Biofilms were sampled once using swabs in pipes connected to each tank. The microbial communities in drain pipes from the weaning section and on-growing section were similar, when considering taxa with high relative abundance (Figure 4B), and most of these taxa were also dominating the communities in biofilms on cable ties (Figure 4A). Although, some variation in relative abundance of taxa were observed in the different sample materials, all drain pipe communities comprised bacterial taxa found in the predominate part of the microbial communities in biofilms and water samples.

### Microbial Communities on Egg and Fish

**FIGURE 5:**
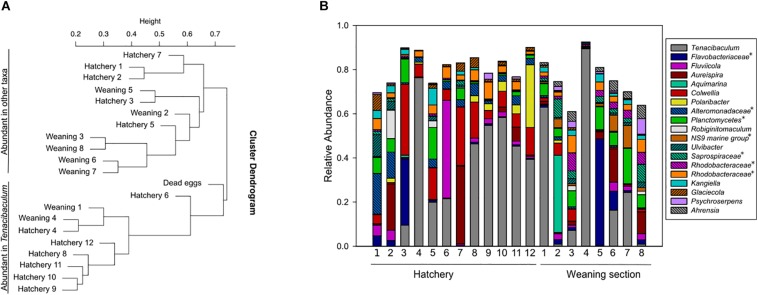
Cluster analysis of amplicon libraries from egg samples **(A)**, and corresponding samples shown as relative abundance **(B)**. The cluster analysis split the dataset in two clusters: one with samples with high diversity and one with samples dominated by *Tenacibaculum*. Eggs from hatchery and incubators in weaning section were found within both clusters **(A)**. Taxa with >5% relative abundance in at least one sample are shown **(B)**. This includes unnamed genera in the Silva database, indicated with an asterisk and named at a higher taxonomic rank.

Eggs were fertilized on site and kept in the hatchery until the incubators were transferred to the weaning section shortly before hatching. The amplicon analysis revealed large variations in the microbial communities on eggs from both locations (Figure 5B). A cluster analysis of all egg samples revealed two clearly separated clusters, where each cluster comprised eggs from both hatchery and weaning section (Figure 5A). Some of the eggs had a high relative abundance of *Tenacibaculum* (<89.5%), a genus with fish pathogenic members. This was also recognizable in the cluster analysis, where eggs in one cluster had microbial communities with high diversity, while eggs in the second cluster were dominated by *Tenacibaculum*. Interestingly, the sample with dead eggs also grouped within the second cluster.

**FIGURE 6:**
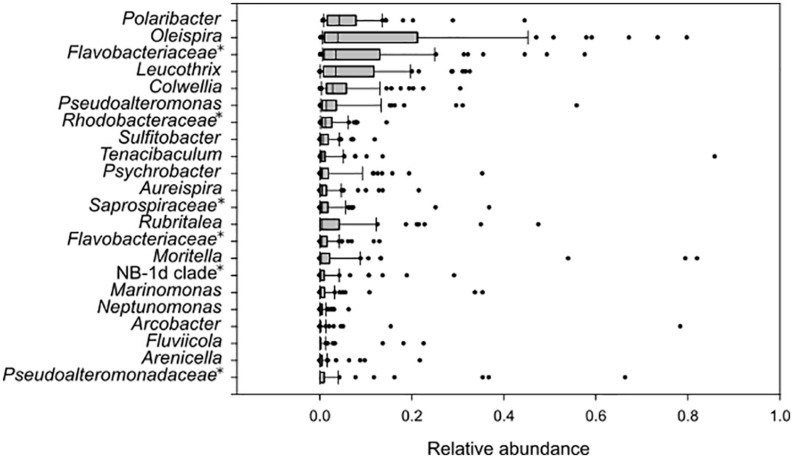
Box plot of amplicon libraries from all fish samples. OTUs with relative abundance above 5% are shown. The gray box includes first and third quartile, and the median. Whiskers show 10th and 90th percentiles, while dots represent outliers in the dataset. Taxa represented as unnamed genera in the Silva database are indicated with an asterisk and named at a higher taxonomic rank.

All fish used in the study appeared healthy at the time of sampling, and there were no reported health problems or infections in the fish farm. The amplicon libraries were based on total DNA from 1 to 5 individuals from each fish tank, sampled from the eight parallel weaning tanks or the four parallel on-growing tanks. The fish were associated with bacterial taxa within *Colwellia*, *Leucothrix*, *Oleispira*, *Polaribacter*, *Pseudoalteromonas*, *Rubritalea*, members of *Saprospiraceae*, *Rhodobacteraceae* and *Flavobacteriaceae*, *Sulfitobacter*, other members of *Pseudoalteromonadaceae*, *Tenacibaculum*, *Psychro- bacter*, *Aureispira*, *Moritella*, NB-1d clade, *Marinomonas*, *Nept- unomonas*, *Arcobacter*, *Fluviicola*, and *Arenicella* which comprised <85.8% relative abundance in total (Figure 6). In general, these taxa were widespread in samples from both weaning- and on-growing sections, but the evenness seemed to be higher in weaning tanks. The taxa *Oleispira*, *Polaribacter*, *Leucothrix*, *Pseudoalteromonas*, *Colwellia*, and members of *Flavobacteriaceae* had consistently high relative abundance (median >1.7%) on sampled fish from both sections, while other taxa had occasional high relative abundances in some samples (Figure 6).

Many of the most abundant taxa found in fish samples were also detected in water or biofilm material from the tanks. However, the taxa *Rubritalea*, members of *Saprospiraceae* and *Rhodobacteraceae*, *Psychrobacter*, *Aureispira*, *Arcobacter*, and *Arenicella* were abundant in fish, but comprised <5% of the microbial communities in the water from fish tanks. Moreover, the taxa *Oleispira*, *Pseudoalteromonas*, *Rubritalea*, *Psychrobacter*, *Moritella*, NB-1d clade, *Marinomonas*, *Arcobacter*, and *Arenicella* were abundant in fish samples, but were found to have relative abundances <5% in biofilms.

### Opportunistic Strains With Pathogenic Potential

Although the sampling was done in a well-maintained fish farm with healthy fish throughout the sampling period, the amplicon analyses of microbial communities revealed the presence of the bacterial taxa *Vibrio*, *Moritella*, and *Tenacibaculum*, which are known to include fish pathogenic members. If monitoring specific pathogenic bacteria within these genera in future studies, it is important to know their relative abundances during healthy conditions. Samples from fish contained both *Tenacibaculum* and *Moritella* with relative abundances >5% (Figure 6). The relative abundances of these taxa were generally low in most fish samples, but occasional peaks of *Moritella* (<21.8%) on fish in weaning tanks, and *Tenacibaculum* (<28.8%) on fish in tanks from both sections were detected. From sampled eggs, only *Tenacibaculum* were prominent (<89.5% relative abundance), while *Moritella* and *Vibrio* comprised <1% of the microbial community. Similarly, the biofilms were mainly abundant in *Tenacibaculum*, while the relative abundances of *Moritella* or *Vibrio* were low (<5%) (Figure 4A). The results of fish tank water analyses showed a higher frequency of samples with pronounced relative abundances of *Tenacibaculum* (<8.0%), *Moritella* (<11.4%) and *Vibrio* (<5.6%) (Figure 3B) compared to the other types of sample material.

## Discussion

The combination of frequent sampling and 16S rRNA gene sequencing provided detailed information about the microbial communities in different sample materials from a flow-through fish farm for lumpfish, and also enabled us to monitor changes in the community compositions over time. This differs from previous studies of lumpfish where bacterial cell counts and cultivation has been the preferred methods, often in studies with bacterial diseases as the main focus (Alarcón et al., 2016; Småge et al., 2016; Norwegian Veterinary Institute [NVI], 2017; Scholz et al., 2018). Pathogenic strains cannot be identified based only on 16S rRNA gene sequencing. Hence, our data does not give detailed information about the distribution of pathogens in the fish-farm. Instead, our study provides a comprehensive overview of the microbial community structures, including genera with known pathogens, present in the different parts of the fish farm and how stable these communities are over time. The fish were healthy throughout the sampling period, and therefore this work represents a baseline study of the regular microbial communities in this type of aquaculture facility under healthy rearing conditions. Our data indicate that high relative abundances of organoheterotrophic non-pathogenic bacteria and generally low relative abundances (<5%) of genera known to include fish pathogens (i.e., *Tenacibaculum*, *Moritella*, and *Vibrio*) are characteristics of microbial communities in healthy fish-farms. This information can be of great value for the interpretation of data from future analyses of microbial communities of fish farms after disease outbreak, or for monitoring specific pathogenic bacteria in order to predict a potential disease outbreak.

The results revealed that the intake water and fish tank water had very different community structures, where all but three samples were found in separate clusters in the PCoA plot. The inlet water was dominated by the marine bacteria SAR86 clade and OCS155 marine group, and the marine archaea “*Candidatus* Nitrosopumilus” and other genera within Marine Group 1. The taxa within each domain are associated with an aerobic and heterotrophic lifestyle (Dupont et al., 2012; West et al., 2016) or ammonia oxidation (Konneke et al., 2005; Swan et al., 2014; Qin et al., 2016), respectively. In contrast, the water in the fish tanks, both weaning- and grow-out tanks, were dominated by the bacterial taxa *Polaribacter*, *Leucothrix*, members of *Flavobacteriaceae*, *Oleispira*, *Colwellia*, *Marinomonas*, *Pseudoalteromonas*, *Tenacibaculum*, *Vibrio*, *Mor- itella*, *Fluviicola*, and *Psychromonas*; where cultivated memb- ers are generally described as organoheterotrophic, aerobic or facultative anaerobic, psychrophilic or mesophilic micro- organisms with origin from marine environments (Yakimov et al., 2003; O’Sullivan et al., 2005; Urakawa, 2014; Bernardet, 2015; Bland and Brock, 2015; Bowman and McMeekin, 2015; Deming and Junge, 2015; Farmer et al., 2015; Sanchez-Amat and Solano, 2015; Staley, 2015; Suzuki, 2015; The Editorial Board, 2015; Dahal and Kim, 2018). The differences in microbial community structures between intake water and fish tank water were probably due to effective UV treatment of the water that greatly reduced the bacterial load, followed by microbial succession in the fish tanks. Low spatial competition and organic rich water in the fish tanks, mainly from dissolved fish feed, fish debris and feces, could provide excellent growth conditions for opportunistic bacteria. However, this could also compromise the health of the fish, if opportunistic pathogens are allowed to develop. Earlier studies have shown that a stable slow-developing community can inhibit blooms of opportunistic bacteria and thereby provide significantly higher survival rates of fry and larvae in tanks with microbial matured water or recirculating aquaculture system (RAS) with a non-pathogenic community, compared to traditional flow-through systems (Skjermo et al., 1997; Attramadal et al., 2012, 2014). This endorses the use of a sand filter for the water connected to the hatchery, which made it enriched in various non-pathogenic taxa. Previous studies also suggest that one key element to achieve a stable microbial community with a probiotic effect is to keep the flow rate of water low enough so that even the slow-growing species within the organoheterotrophic non-pathogenic population are retained in the fish tanks without getting flushed away (Attramadal et al., 2012; De Schryver and Vadstein, 2014). Furthermore, studies from aquaculture systems have demonstrated the importance of organic matter removal in order to maintain a stable microbial community in the water (Cripps and Bergheim, 2000; Leonard et al., 2000; De Schryver and Vadstein, 2014). In our study, the microbial analyses showed high similarities between parallel fish tanks and stable microbial community structures over time in the water. Stable microbial communities and healthy fish in the fish farm could indicate that the flow rate in the fish tanks were adequate for establishment of slow-growing microorganisms, in addition to the fast-growing non-pathogenic organoheterotrophic bacteria, that promote healthy rearing conditions without high relative abundances of taxa with pathogenic members. In addition, good routines for daily maintenance at the fish farm, including regular removal of organic matter, seem to be vital for a beneficial microbial community structure over time.

Despite daily maintenance, biofilm formation on surfaces in the tanks and pipes is inevitable. The main differences included the taxa *Leucothrix*, *Aureispira*, and *Winogradskyella*, which had higher relative abundances in biofilms compared to water, probably due to their physical properties of gliding or wave-like motility that could support biofilm formation (Hosoya et al., 2006; Bland and Brock, 2015; Nedashkovskaya and Mikhailov, 2015). Many marine pathogenic bacteria use acetylated homoserine lactone (AHL) in quorum sensing for cell-to-cell communication, and this mechanism is used by *Tenacibaculum* to shift between biofilm and a pelagic life style (Bruhn et al., 2005; Romero et al., 2010). The shift is suggested to be important for interactions between bacteria and eukaryotes (Romero et al., 2010), and it is hypothesized that *Tenacibaculum* might establish in biofilms and later initiate infection through quorum sensing. In this study, however, the biofilms were not dominated by filamentous *Tenacibaculum*, but by non-pathogenic bacteria instead. Hence, the biofilm does not seem to support high relative abundances of taxa with pathogenic potential during periods with healthy rearing conditions.

The dominating taxa in fish were also detected in fish tank water and biofilms, but other taxa had also occasional high relative abundances in some samples. In general, the microbial communities associated with fish were more variable compared to water and biofilm, probably due to their individual differences in genetic pool and health status. The fish looked healthy upon sampling, but in some fish high relative abundances of *Tenacibaculum* and *Moritella* were detected, which are known to include pathogenic members associated with fish disease (Wakabayashi et al., 1986; Lunder et al., 2000; Avendano-Herrera et al., 2006; Habib et al., 2014). These genera were also detected in the water and biofilms, with high relative abundances in some samples. Furthermore, sequences classified as *Vibrio*, a genus with pathogenic members that could cause infection on lumpfish (Scholz et al., 2018), was detected in all types of sample material, but had in general higher relative abundances in water samples, compared to fish and biofilms. Hence, genera known to contain pathogenic strains were occasionally detected with high relative abundances during periods with healthy fish. This could indicate that the taxa present during normal rearing conditions were represented by non-pathogenic strains, or that certain requirements need to be fulfilled for a disease outbreak to occur. Future studies using strain-specific approaches are required to reveal this. High relative abundances of *Tenacibaculum* were also detected on eggs covered with white spots or white layer, which indicated that the eggs were dead or damaged. These eggs seemed to have lower hatching rates (pers. comm. B. Nordhus, May 2017). Previous studies have shown that species of *Tenacibaculum* can cause high mortalities in halibut eggs and larvae (Bergh et al., 1992; Hansen et al., 1992). Furthermore, studies on the filamentous bacterium *Leucothrix* on eggs from cod, halibut and lobster revealed that the microbe forms dense biofilms on the eggs, which consume oxygen and hence deprive oxygen-flow to the eggs (Hansen and Olafsen, 1989; Sadusky and Bullis, 1994). The *Leucothrix* biofilm is thick enough to be visible to the naked eye, and is often mistaken as a fungal infection on the eggs. These biofilms could therefore result in poor quality fry due to premature hatching or low hatching rate. A similar mechanism could occur by the filamentous *Tenacibaculum* in our egg samples, but this will have to be confirmed in future studies. Some of the eggs that appeared healthy had also high relative abundances of *Tenacibaculum.* This could indicate that opportunistic bacteria, such as *Tenacibaculum*, could be transferred from the hatchery to the weaning tanks, and thereby spread a potentially unwanted bacterium to the surrounding water or fry.

## Conclusion

In conclusion, the 16S rRNA gene sequencing seems to be a cost- and time-effective approach for monitoring the microbial communities present at different sites in fish farms. This study was conducted during regular operational farming conditions with no disease outbreak, and describes the typical microbial community structures in this type of rearing facility, which can be important knowledge for comparative studies of different fish farms or different operational conditions. The results showed that the UV-treated water was enriched in non-pathogenic bacteria in the fish tanks. Samples of water, biofilms and fish were similar when considering taxa with high relative abundances (>5%), and showed that the communities were stable over time. However, samples with high relative abundances of genera with pathogenic members, such as *Moritella*, *Tenacibaculum*, and *Vibrio*, were detected during regular operational farming conditions. Some of the eggs were dominated by *Tenacibaculum*, which might be associated with low hatching rate.

## Ethics Statement

The study used fish collected from ordinary breeding and production cycle at the fish farm, which are not under the act of animal ethic legislation concerning use of animals in Norway. Therefore, no ethical committee is required. Sampled fish were humanely euthanized according to the Norwegian law, as stated in the manuscript.

## Author Contributions

IR did sampling at the location, performed DNA extractions and amplicon library preparations, did the ecological analyses and interpretation of data, and wrote the manuscript. KD did sampling at the location, was involved in the interpretation of the data, and revised the manuscript. HD provided bioinformatics tools for sorting, cleaning, and taxonomical classification of the amplicon reads, and also helped in the analysis and interpretation of the data, and revised the manuscript. HW revised the manuscript.

## Conflict of Interest Statement

The authors declare that the research was conducted in the absence of any commercial or financial relationships that could be construed as a potential conflict of interest.

## Footnotes

1 https://drive5.com/uchime/uchime_download.html

## Supplementary Material

FIGURE S1 Hierarchical cluster analysis of allsamples at genus level. The Hierarchical cluster analysis wasperformed in the Community ecology “vegan” package in R (version3.5) using the ward algorithm (ward.D2), based on a Bray–Curtis distance matrix of the data. Samples were given an individual identification number, which is listed in Supplementary Table S1.

TABLE S1 Sample overview.

TABLE S2 OTU table of all samples.

TABLE S3 Distance matrix of all samples based on Bray–Curtis dissimilarities.

## References

[B1] Alarcón M. Gulla S. Røsæg M. V. Rønneseth A. Wergeland H. Poppe T. T. (2016). Pasteurellosis in lumpsucker *Cyclopterus lumpus*, farmed in Norway. *J. Fish Dis.* 39 489–495. 25828053 10.1111/jfd.12366

[B2] Anderson M. J. (2001). A new method for non-parametric multivariate analysis of variance. *Austral Ecol.* 26 32–46. 10.1111/j.1442-9993.2001.01070.pp.x

[B3] Anderson M. J. (2006). Distance-based tests for homogeneity of multivariate dispersions. *Biometrics* 62 245–253. 10.1111/j.1541-0420.2005.00440.x 16542252

[B4] Anderson M. J. Walsh D. C. I. (2013). PERMANOVA, ANOSIM, and the Mantel test in the face of heterogeneous dispersions: What null hypothesis are you testing? *Ecol. Monogr.* 83 557–574. 10.1890/12-2010.1

[B5] Attramadal K. J. K. Salvesen I. Xue R. Øie G. Størseth T. R. Vadstein O. (2012). Recirculation as a possible microbial control strategy in the production of marine larvae. *Aquac. Eng.* 46 27–39. 10.1016/j.aquaeng.2011.10.003

[B6] Attramadal K. J. K. Truong T. M. H. Bakke I. Skjermo J. Olsen Y. Vadstein O. (2014). RAS and microbial maturation as tools for K-selection of microbial communities improve survival in cod larvae. *Aquaculture* 432 483–490. 10.1016/j.aquaculture.2014.05.052

[B7] Avendano-Herrera R. Toranzo A. E. Magarinos B. (2006). Tenacibaculosis infection in marine fish caused by *Tenacibaculum maritimum*: A review. *Dis. Aquat. Organ.* 71 255–266. 10.3354/dao071255 17058606

[B8] Benjamini Y. Hochberg Y. (1995). Controlling the false discovery rate - a practical and powerful approach to multiple testing. *J. R. Stat. Soc. B* 57 289–300. 10.1111/j.2517-6161.1995.tb02031.x

[B9] Bergh Ø Hansen G. H. Taxt R. E. (1992). Experimental infection of eggs and yolk sac larvae of halibut, *Hippoglossus hippoglossus* L. *J. Fish Dis.* 15 379–391. 10.1111/j.1365-2761.1992.tb01237.x

[B10] Bernardet J. F. (2015). “Flavobacteriaceae,” in *Bergey’s Manual of Systematics of Archaea and Bacteria*, eds Whitman W. B. Rainey F. Kämpfer P. Trujillo M. Chun J. DeVos P. (Hoboken, NJ: Wiley), 853–871.

[B11] Berry D. Ben Mahfoudh K. Wagner M. Loy A. (2011). Barcoded primers used in multiplex amplicon pyrosequencing bias amplification. *Appl. Environ. Microb.* 77 7846–7849. 10.1128/AEM.05220-11 21890669 PMC3209180

[B12] Bland J. A. Brock T. D. (2015). “Leucothrix,” in *Bergey’s Manual of Systematics of Archaea and Bacteria*, eds Whitman W. B. Rainey F. Kämpfer P. Trujillo M. Chun J. DeVos P. (Hoboken, NJ: Wiley), 25–37.

[B13] Bowman J. P. McMeekin T. A. (2015). “Pseudoalteromonas,” in *Bergey’s Manual of Systematics of Archaea and Bacteria*, eds Whitman W. B. Rainey F. Kämpfer P. Trujillo M. Chun J. DeVos P. (Hoboken, NJ: Wiley), 239–261.

[B14] Bruhn J. B. Dalsgaard I. Nielsen K. F. Buchholtz C. Larsen J. L. Gram L. (2005). Quorum sensing signal molecules (acylated homoserine lactones) in gram-negative fish pathogenic bacteria. *Dis. Aquat. Organ.* 65 43–52. 10.3354/dao065043 16042042

[B15] Caporaso J. G. Kuczynski J. Stombaugh J. Bittinger K. Bushman F. D. Costello E. K. (2010). QIIME allows analysis of high-throughput community sequencing data. *Nat. Methods* 7 335–336. 20383131 10.1038/nmeth.f.303 PMC3156573

[B16] Cripps S. J. Bergheim A. (2000). Solids management and removal for intensive land-based aquaculture production systems. *Aquac. Eng.* 22 33–56. 10.1016/s0144-8609(00)00031-5

[B17] Dahal R. H. Kim J. (2018). *Fluviicola kyonggii* sp. nov., a bacterium isolated from forest soil and emended description of the genus *Fluviicola*. *Int. J. Syst. Evol. Microbiol.* 68 1885–1889. 10.1099/ijsem.0.002759 29648526

[B18] Dahle S. W. Hagemann A. Attramadal K. J. Kjørsvik E. Bardal T. (2017). *AnnVkvalitet og Startfôring av Rognkjeks.* Trondheim: SINTEF Fiskeri og havbruk, 39.

[B19] De Schryver P. Vadstein O. (2014). Ecological theory as a foundation to control pathogenic invasion in aquaculture. *ISME J.* 8 2360–2368. 10.1038/ismej.2014.84 24892581 PMC4260705

[B20] Deming J. W. Junge K. (2015). “Colwellia,” in *Bergey’s Manual of Systematics of Archaea and Bacteria*, eds Whitman W. B. Rainey F. Kämpfer P. Trujillo M. Chun J. DeVos P. (Hoboken, NJ: Wiley), 1257–1369.

[B21] Dupont C. L. Rusch D. B. Yooseph S. Lombardo M.-J. Richter A. R. Valas R. (2012). Genomic insights to SAR86, an abundant and uncultivated marine bacterial lineage. *ISME J.* 6 1186–1199. 10.1038/ismej.2011.189 22170421 PMC3358033

[B22] Edgar R. C. (2010). Search and clustering orders of magnitude faster than BLAST. *Bioinformatics* 26 2460–2461. 10.1093/bioinformatics/btq461 20709691

[B23] Edgar R. C. (2013). UPARSE: Highly accurate OTU sequences from microbial amplicon reads. *Nat. Methods* 10 996–998. 10.1038/nmeth.2604 23955772

[B24] Farmer J. Janda M. J. Brenner F. W. Cameron D. N. Birkhead K. M. (2015). “Vibrio,” in *Bergey’s Manual of Systematics of Archaea and Bacteria*, eds Whitman W. B. Rainey F. Kämpfer P. Trujillo M. Chun J. DeVos P. (Hoboken, NJ: Wiley), 77–156.

[B25] Habib C. Houel A. Lunazzi A. Bernardet J.-F. Olsen A. B. Nilsen H. (2014). Multilocus sequence analysis of the marine bacterial genus *Tenacibaculum* suggests parallel evolution of fish pathogenicity and endemic colonization of aquaculture systems. *Appl. Environ. Microb.* 80 5503–5514. 10.1128/AEM.01177-14 24973065 PMC4136090

[B26] Hansen G. H. Bergh O. Michaelsen J. Knappskog D. (1992). *Flexibacter ovolyticus* sp. nov., a pathogen of eggs and larvae of *Atlantic halibut*, *Hippoglossus hippoglossus L*. *Int. J. Syst. Bacteriol.* 42 451–458. 10.1099/00207713-42-3-451 1503974

[B27] Hansen G. H. Olafsen J. A. (1989). Bacterial colonization of cod (*Gadus morhua* L.) and halibut (*Hippoglossus hippoglossus*) eggs in marine aquaculture. *Appl. Environ. Microbiol.* 55 1435–1446. 16347937 10.1128/aem.55.6.1435-1446.1989 PMC202883

[B28] Hosoya S. Arunpairojana V. Suwannachart C. Kanjana-Opas A. Yokota A. (2006). *Aureispira marina* gen. nov., sp. nov., a gliding, arachidonic acid-containing bacterium isolated from the southern coastline of Thailand. *Int. J. Syst. Evol. Microbiol.* 56 2931–2935. 10.1099/ijs.0.64504-0 17159001

[B29] Imsland A. K. Reynolds P. Eliassen G. Hangstad T. A. Foss A. Vikingstad E. (2014). The use of lumpfish (*Cyclopterus lumpus* L.) to control sea lice (*Lepeophtheirus salmonis Krøyer*) infestations in intensively farmed Atlantic salmon (*Salmo salar L*.). *Aquaculture* 424–425 18–23. 10.1016/j.aquaculture.2013.12.033

[B30] Klindworth A. Pruesse E. Schweer T. Peplies J. Quast C. Horn M. (2013). Evaluation of general 16S ribosomal RNA gene PCR primers for classical and next-generation sequencing-based diversity studies. *Nucleic Acids Res.* 41:e1. 10.1093/nar/gks808 22933715 PMC3592464

[B31] Konneke M. Bernhard A. E. de la Torre J. R. Walker C. B. Waterbury J. B. Stahl D. A. (2005). Isolation of an autotrophic ammonia-oxidizing marine archaeon. *Nature* 437 543–546. 10.1038/nature03911 16177789

[B32] Leonard N. Blancheton J. P. Guiraud J. P. (2000). Populations of heterotrophic bacteria in an experimental recirculating aquaculture system. *Aquac. Eng.* 22 109–120. 19508294

[B33] Lunder T. Sorum H. Holstad G. Steigerwalt A. G. Mowinckel P. Brenner D. J. (2000). Phenotypic and genotypic characterization of *Vibrio viscosus* sp. nov. and *Vibrio wodanis* sp. nov. isolated from Atlantic salmon (*Salmo salar*) with ‘winter ulcer’. *Int. J. Syst. Evol. Microbiol.* 50 427–450. 10.1099/00207713-50-2-427 10758846

[B34] Murtagh F. Legendre P. (2014). Ward’s hierarchical agglomerative clustering method: Which algorithms implement ward’s criterion? *J. Classif.* 31 274–295. 10.1007/s00357-014-9161-z

[B35] Nedashkovskaya O. I. Mikhailov V. V. (2015). “Winogradskyella,” in *Bergey’s Manual of Systematics of Archaea and Bacteria*, eds Whitman W. B. Rainey F. Kämpfer P. Trujillo M. Chun J. DeVos P. (Hoboken, NJ: Wiley), 995–1016.

[B36] Norwegian Directorate of Fisheries (2017). *Statistikk for Akvakultur 2017.* Bergen: Norwegian Directorate of Fisheries.

[B37] Norwegian Veterinary Institute [NVI] (2017). *The Health Situation in Norwegian Aquaculture 2016*, eds Hjeltnes B. Bornø G. Jansen M. D. Haukaas A. Walde C. S., Oslo: Norwegian Veterinary Institute.

[B38] Oksanen J. Blanchet F. G. Friendly M. Kindt R. Legendre P. McGlinn D. (2018). *vegan: Community Ecology Package, R Package Version 2.5-2.*

[B39] O’Sullivan L. A. Rinna J. Humphreys G. Weightman A. J. Fry J. C. (2005). *Fluviicola taffensis* gen. nov., sp. nov., a novel freshwater bacterium of the family *Cryomorphaceae* in the phylum ‘*Bacteroidetes*’. *Int. J. Syst. Evol. Microbiol.* 55 2189–2194. 10.1099/ijs.0.63736-0 16166730

[B40] Ovreas L. Forney L. Daae F. L. Torsvik V. (1997). Distribution of bacterioplankton in meromictic Lake Saelenvannet, as determined by denaturing gradient gel electrophoresis of PCR-amplified gene fragments coding for 16S rRNA. *Appl. Environ. Microb.* 63 3367–3373. 9292986 10.1128/aem.63.9.3367-3373.1997 PMC168642

[B41] Powell A. Treasurer J. W. Pooley C. L. Keay A. J. Lloyd R. Imsland A. K. (2018). Use of lumpfish for sea−lice control in salmon farming: Challenges and opportunities. *Rev. Aquac.* 10 683–702. 10.1111/raq.12194

[B42] Qin W. Martens-Habbena W. Kobelt J. N. Stahl D. A. (2016). “*Candidatus* Nitrosopumilus,” in *Bergey’s Manual of Systematics of Archaea and Bacteria*, eds Whitman W. B. Rainey F. Kämpfer P. Trujillo M. Chun J. DeVos P. (Hoboken, NJ: Wiley), 8818–8827.

[B43] R Core Team (2018). *R: A Language and Environment for Statistical Computing.* Vienna: R Foundation for Statistical Computing.

[B44] Romero M. Avendano-Herrera R. Magarinos B. Camara M. Otero A. (2010). Acylhomoserine lactone production and degradation by the fish pathogen *Tenacibaculum maritimum*, a member of the Cytophaga-Flavobacterium-Bacteroides (CFB) group. *FEMS Microbiol. Lett.* 304 131–139. 10.1111/j.1574-6968.2009.01889.x 20377642

[B45] Sadusky T. J. Bullis R. A. (1994). Experimental disinfection of lobster eggs infected with *Leucothrix mucor*. *Biol. Bull.* 187 254–255. 10.1086/bblv187n2p254 7811807

[B46] Sanchez-Amat A. Solano F. (2015). “Marinomonas,” in *Bergey’s Manual of Systematics of Archaea and Bacteria*, eds Whitman W. B. Rainey F. Kämpfer P. Trujillo M. Chun J. DeVos P. (Hoboken, NJ: Wiley), 3057–3066.

[B47] Scholz F. Glosvik H. Marcos-López M. (2018). “Cleaner fish health,” in *Cleaner Fish Biology and Aquaculture Applications*, ed. Treasurer J. (Sheffield: 5M Publishing), 221–257.

[B48] Skjermo J. Salvesen I. Øie G. Olsen Y. Vadstein O. (1997). Microbially matured water: A technique for selection of a non-opportunistic bacterial flora in water that may improve performance of marine larvae. *Aquac. Int.* 5 13–28. 10.1007/bf02764784

[B49] Småge S. B. Frisch K. Brevik ØJ. Watanabe K. Nylund A. (2016). First isolation, identification and characterisation of *Tenacibaculum maritimum* in Norway, isolated from diseased farmed sea lice cleaner fish *Cyclopterus lumpus* L. *Aquaculture* 464 178–184. 10.1016/j.aquaculture.2016.06.030

[B50] Staley J. T. (2015). “Polaribacter,” in *Bergey’s Manual of Systematics of Archaea and Bacteria*, eds Whitman W. B. Rainey F. Kämpfer P. Trujillo M. Chun J. DeVos P. (Hoboken, NJ: Wiley), 283–304.

[B51] Suzuki M. (2015). “Tenacibaculum,” in *Bergey’s Manual of Systematics of Archaea and Bacteria*, eds Whitman W. B. Rainey F. Kämpfer P. Trujillo M. Chun J. DeVos P. (Hoboken, NJ: Wiley), 793–800.

[B52] Swan B. K. Chaffin M. D. Martinez-Garcia M. Morrison H. G. Field E. K. Poulton N. J. (2014). Genomic and metabolic diversity of Marine Group I *Thaumarchaeota* in the mesopelagic of two subtropical gyres. *PLoS One* 9:e95380. 10.1371/journal.pone.0095380 24743558 PMC3990693

[B53] The Editorial Board (2015). “Psychromonas,” in *Bergey’s Manual of Systematics of Archaea and Bacteria*, eds Whitman W. B. Rainey F. Kämpfer P. Trujillo M. Chun J. DeVos P. (Hoboken, NJ: Wiley), 527–531.

[B54] Treasurer J. (ed.) (2018). “An introduction to sea lice and the rise of cleaner fish,” in *Cleaner Fish Biology and Aquaculture Applications* (Sheffield: 5M Publishing), 3–25.

[B55] Urakawa H. (2014). “The family *Moritellaceae*,” in *The Prokaryotes: Gammaproteobacteria*, eds Rosenberg E. Delong E. F. Lory S. Stackebrandt E. Thompson F. (Berlin: Springer-Verlag), 477–489. 10.1007/978-3-642-38922-1_227

[B56] Wakabayashi H. Hikida M. Masumura K. (1986). *Flexibacter maritimus* sp. nov., a pathogen of marine fishes. *Int. J. Syst. Bacteriol.* 36 396–398. 10.1099/00207713-36-3-396

[B57] West N. J. Lepere C. Manes C. L. Catala P. Scanlan D. J. Lebaron P. (2016). Distinct spatial patterns of SAR11, SAR86, and *Actinobacteria* diversity along a transect in the ultra-oligotrophic south Pacific Ocean. *Front. Microbiol.* 7:234. 10.3389/fmicb.2016.00234 27014192 PMC4781884

[B58] Yakimov M. M. Giuliano L. Gentile G. Crisafi E. Chernikova T. N. Abraham W.-R. (2003). *Oleispira antarctica* gen. nov., sp. nov., a novel hydrocarbonoclastic marine bacterium isolated from Antarctic coastal sea water. *Int. J. Syst. Evol. Microbiol.* 53 779–785. 10.1099/ijs.0.02366-0 12807200

