# Supplementary figures and images for: Microbial Communities in a Flow-Through Fish Farm for Lumpfish (Cyclopterus lumpus L.) During Healthy Rearing Conditions
Source: Front Microbiol. 2019 Jul 12;10:1594. doi: 10.3389/fmicb.2019.01594 (PMC6640156; doi:10.3389/fmicb.2019.01594)

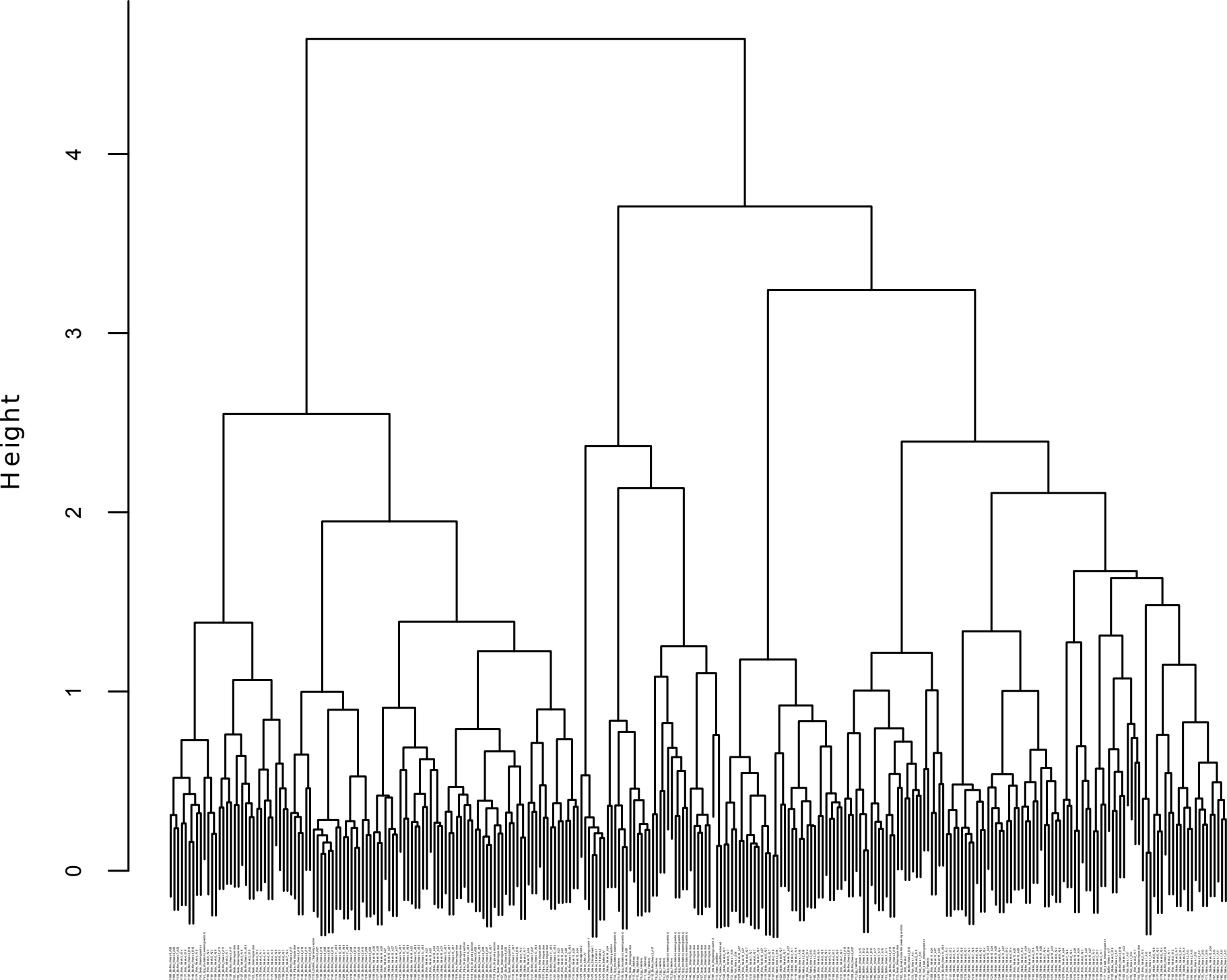

Supplement: FIGURE S1 — Hierarchical cluster analysis of allsamples at genus level. The Hierarchical cluster analysis wasperformed in the Community ecology “vegan” package in R (version3.5) using the ward algorithm (ward.D2), based on a Bray–Curtis distance matrix of the data. Samples were given an individual identification number, which is listed in Supplementary Table S1. [file Image_1.pdf]
